# Effects of Ashwagandha-derived silver nanoparticles on pre- and post-thaw semen quality in bulls

**DOI:** 10.3389/fvets.2025.1709707

**Published:** 2026-01-28

**Authors:** Arushi Kanwar, Meenakshi Virmani, Kartik Chaudhary, Sandeep Kumar, Preeti Lakhani, Rajesh Kumar, Neeraj Panihar

**Affiliations:** 1 Department of Veterinary Physiology and Biochemistry, Lala Lajpat Rai University of Veterinary and Animal Sciences, Hisar, Haryana, India; 2 Forest Department-Wildlife Wing, Paonta Sahib, Himachal Pradesh, India; 3 Department of Veterinary Gynaecology and Obstetrics, Lala Lajpat Rai University of Veterinary and Animal Sciences, Hisar, Haryana, India

**Keywords:** antibacterial, antioxidant, Ashwagandha, nanoparticle, sliver

## Abstract

This study aimed to evaluate the effects of silver nanoparticle (AgNP) supplementation on bovine semen quality, with a focus on determining whether green-synthesized AgNPs derived from ethanolic *Withania somnifera* (ashwagandha) extract could mitigate cryopreservation-induced damage. Bovine semen samples were supplemented with AgNPs at concentrations of 10, 20, 30, 40, and 50 μg/mL. The effects on semen quality were assessed by measuring antioxidant activity, sperm motility, morphological abnormalities, acrosome integrity, and lipid peroxidation, both in fresh semen and after cryopreservation. AgNP supplementation led to a dose-dependent increase in antioxidant activity. Among the concentrations tested, 20 μg/mL produced the most significant improvements in sperm motility and a reduction in morphological abnormalities in fresh semen. This concentration also enhanced acrosome integrity and decreased lipid peroxidation, indicating protection against oxidative stress during cryopreservation. While post-thaw motility showed limited differences, pre-freezing kinematic parameters improved with AgNP supplementation, suggesting enhanced sperm quality.Green-synthesized AgNPs, particularly at 20 μg/mL, have the potential to improve bovine semen quality and reproductive performance by reducing oxidative stress and supporting sperm function. These findings suggest AgNPs as promising additives for improving livestock reproduction, with additional research needed to explore broader applications in reproductive biology and agricultural sciences.

## 1 Introduction

Sperm cryopreservation has been reported to induce an increase in plasma membrane fluidity permeability, overproduction of reactive oxygen species (ROS), reduction of acrosome integrity, impairment of mitochondrial membrane potential and a lower sperm motility in bull (1, 2), buffalo (3, 4), buck (5), ram (6), and red deer (7). Reduced metabolic activity, loss of cytoplasmic proteins, membrane-bound proteins, enzymes and other cellular components are some of the insults to sperm cells that may cause defects in sperm proteins and may impair sperm motility, fertilization and immediate post fertilization processes (8). The advancement of nanotechnology has introduced innovative approaches across various scientific fields, including reproductive biology. Among the diverse nanomaterials, green synthesized silver nanoparticles (AgNPs) have garnered significant attention due to their unique physicochemical properties and potential applications in improving reproductive outcomes. Conventional sperm cryopreservation methods, while essential for artificial insemination and genetic preservation, are associated with significant challenges, including oxidative stress, membrane damage, and reduce sperm motility post-thaw (1, 6). These limitations necessitate the exploration of novel protective agents to enhance sperm viability and functionality. Oxidative stress during cryopreservation arises from an imbalance between reactive oxygen species (ROS) production and the antioxidant defense system, leading to lipid peroxidation, DNA fragmentation, and impaired sperm function (9). The primary sources of ROS in semen include leukocytes, morphologically defective spermatozoa, and cold shock exposure during freezing (10, 33). Despite the use of conventional antioxidants, sperm damage during cryopreservation remains a critical issue. Therefore, there is a need to explore alternative antioxidant strategies that not only mitigate ROS damage but also enhance post-thaw sperm quality. Moreover, the antioxidative protection of the cryopreserved semen was strengthened by supplementation of plant extracts (47, 48). Additionally, research suggests that AgNPs enhance bioavailability and potency compared to raw plant extracts (49).

However, despite the potential of AgNPs in biomedical applications, their impact on sperm cryopreservation remains largely unexplored. LPO is a major form of oxidative damage to sperm membranes, which are rich in polyunsaturated fatty acids. *Withaferin* and other withanolides suppress LPO, protecting the structural integrity of sperm cells. Ashwagandha treatment has been shown to restore and increase the activity of key endogenous antioxidant enzymes within the seminal plasma, such as superoxide dismutase (SOD) and catalase. It also increases the levels of non-enzymatic antioxidants like vitamins A, C, and E, and glutathione (GSH), which are crucial for neutralizing reactive oxygen species (ROS). By lowering ROS concentrations, withaferin A helps reduce the rate of sperm cell death (apoptosis), leading to a higher concentration of viable sperm. The exact mechanisms through which AgNPs influence sperm integrity, motility, and oxidative stress levels require further investigation.

## 2 Material and methods

### 2.1 Preparation and characterization of silver nanoparticles

A 0.2% ethanolic extract of ashwagandha root powder was prepared by stirring at 40 °C for 1 h, followed by centrifugation at 5,000 rpm to collect the supernatant. A 6.5 mM silver sulfate solution was prepared, and 5 mL of the extract was added dropwise to 10 mL of the solution while stirring at 60 °C, with the pH adjusted to 9 using 1N NaOH. The mixture was stirred further, and the synthesized. nanoparticles were centrifuged at 12,000 rpm, washed thrice with distilled water, and dried at 60–70 °C for collection. Characterization included UV-Vis spectrophotometry, dynamic light scattering for particle size (Zetasizer Nano ZS), TEM (JEM-2100) and SEM (ZEISS EVO MA10) for morphology, XRD (Bruker Axes D-8) for crystal structure, and Raman spectroscopy (Airix) to analyze vibrational modes and molecular composition.

### 2.2 Antioxidant activity by DPPH (2,2-diphenyl-1-picrylhydrazyl) method

The antioxidant activity of the nanoparticles was evaluated using the DPPH (2,2-diphenyl 1- picrylhydrazyl) radical scavenging assay, following the method outlined by Shimada et al. (50). Using ascorbic acid in methanol as standard. Both the biosynthesized silver nanoparticles (AgNPs and the ethanolic extract were assessed for their antioxidant capabilities. Various concentrations of the nanoparticles (10, 20, 30, 40, 60, 80 μg/mL) were combined with 200 μL of a methanolic DPPH solution (0.1 mM). The mixtures were incubated for 30 min at room temperature, after which the absorbance was measured at 517 nm. The percentage inhibition of the antioxidant activity was calculated using the formula: [(Ao – Ae)/Ao] x 100 (Ao = absorbance of control; Ae absorbance of extract).

### 2.3 Selection of optimum concentration of green silver nanoparticles for supplementation in semen

Mature cattle bulls, aged 4–5 years and weighing between 350 and 450 kg, were selected for this study. These bulls were healthy, exhibited good libido, and were housed individually in semi-open sheds at the Animal Farm, Department of Animal Genetics and Breeding, College of Veterinary Sciences, Lala Lajpat Rai University of Veterinary and Animal Sciences, Hisar. They were provided with a balanced breeding bull diet and had *ad libitum* access to drinking water.

Five ejaculates were collected from each of the two fertile bulls (*n* = 10) using a sterilized artificial vagina at weekly intervals, following the protocol by Barszcz et al. (46). Each bull was allowed at least one false mount before collection. Immediately after semen collection, each ejaculate was assessed for volume, motility, and concentration as per Murphy et al. (45). Fresh semen samples with motility ≥ 70% were diluted with BioXcell (Ref. 016218-006584-018617) freezing medium having an antibioticmix of Lincomycin, Spectinomycin, Gentamycin and Tylosin but no egg ypolk or milk, which is an Animal protein-free, vegetal protein-based extender for frozen bovine semen, and then divided into seven groups. The first group served as the control, while the other groups were supplemented with nanoparticles at concentrations of 10, 20, 30, and 40 μg/mL of semen with reference from work done by Yousef et al. (11) and Kanwar et al. (12). The semen samples were incubated at 37 °C and evaluated for motility parameters.

Sperm kinetics and motility, as well as morphological anomalies, were assessed using the computer- assisted sperm analysis (CASA) system (IVOS II™ Clinical, Hamilton-Thorne Biosciences, Beverly, MA, USA) with a frame rate of 60 Hz and an analysis grid of 10 × negative phase contrast for motility or 40 × brightfield for morphology. For each sample, five optical fields were selected from each chamber of the eight chambered Leja slide (depth 20 μm) with a Stage Temperature of 37 °C.

### 2.4 Freezing of semen supplemented with optimized concentration of silver nanoparticles

A total of 37 semen ejaculates were collected twice weekly from two mature cattle bulls using a sterilized artificial vagina. The semen samples were evaluated for volume, sperm concentration, and percentage of motile spermatozoa. Sperm motility was subjectively assessed under a phase-contrast microscope equipped with a warm stage (37 °C) at 200X magnification. Only 31 ejaculates with ≥70% sperm motility were selected for cryopreservation. The semen was diluted with an extender to achieve a sperm concentration of 80 million/mL and then divided into two aliquots. One aliquot served as the control (Group 1), while the other aliquot (Group 2) was supplemented with 20 μg/mL of silver nanoparticles synthesized using ethanolic extracts of ashwagandha Cryopreservation of sperm involves a series of steps including temperature reduction, cell dehydration, freezing, and subsequent storage (13). The semen sample was placed into 0.25 ml French straws using an automatic straw Filling device. The straws were allowed to equilibrate at 4 °C in a cold cabinet for 4 h before being moved to a biological freezer where they were frozen in liquid nitrogen vapor. The freezing rate used in the biological freezer was −19 °C per minute, ultimately reaching −141 °C in seven minutes. Following this, the straws were preserved in liquid nitrogen.

### 2.5 Evaluation of semen supplemented with optimized concentration of silver nanoparticles

#### 2.5.1 Estimation of sperm kinetics and motility by CASA

The sperm kinetics and motility as well as morphological anomalies were assessed using the computer assisted sperm analyzer (CASA) system as described in pre-freezing stage. For post-freezing analysis, two semen straws from each group (*n* = 10) were thawed in a water bath at 37 °C for 30 s. The contents of straws were transferred to 1.5 mL tubes maintained in a dry bath at 37 °C and assessed using CASA.

#### 2.5.2 Estimation of sperm livability

Eosin-nigrosin stain was prepared by dissolving 0.67 g of eosin and 10 g of nigrosin in 100 mL of distilled water and the stain was filtered using Whatmann filter paper. Viability of sperm was detected by eosin- nigrosin staining method as described by Campbell et al. (44). For this procedure, eosin-nigrosin stain was slightly warmed at 37 °C in hot air oven for 30 min. The frozen-thawed semen mixed with eosin-nigrosin stain was placed on pre-warmed clean grease free glass slide and a thin smear was prepared. The smear was air dried, and the slides were examined under microscope (1000X), first starting with low magnification (400X) in order to get an overview of the stained sperms. Live spermatozoa remained unstained while dead spermatozoa took pink-red stain against blue-black background, partially stained sperms counted as dead. Approximately 200 sperms from different fields were counted and percent live-dead sperm was calculated.

#### 2.5.3 Estimation of acrosome integrity

Giemsa stain was employed to assess the percentage of intact acrosomes, following the method described by Watson and Martin (43). The stock Giemsa stain was prepared by thoroughly mixing 1 g of Giemsa stain with 66 mL of methanol and 60 mL of glycerol. This mixture was maintained at 37 °C for 7 days, being stirred once every 24 h. After the 7-day period, the solution was filtered and stored at 5 °C. Prior to use, the solution was brought to room temperature (22–25 °C). The working Giemsa stain was prepared by combining 3 mL of the stock Giemsa solution with 2 mL of Sorenson's buffer and 45 mL of distilled water. Sorenson's phosphate buffer was made by mixing two solutions in a 1:1 ratio to achieve a pH of 6.8 at 20 °C: - Solution A (M/15 Na2PO4 buffer): 9.47 gm of anhydrous disodium phosphate (Na2PO4) dissolved in 1 liter of distilled water. - Solution B (M/15KH2PO4 buffer):^**^ 9.09 gm of anhydrous potassium dihydrogen phosphate (KH2PO4) dissolved in 1 liter of distilled water.

To determine the percentage of intact acrosomes, a thin smear of extended semen was prepared on a clean and sterilized glass slide and dried on a warm stage (37 °C) for 2–3 min. The smear was then fixed in a neutral formalin saline solution for 15 min and gently rinsed with running tap water. The prepared smear was stained for 40 min in the working Giemsa stain solution. The degree of acrosome integrity was evaluated using a phase-contrast microscope under an oil immersion lens at 100X magnification.

#### 2.5.4 Lipid peroxidation assay

The method of Ohkawa et al. (42) was used to determine malondialdehyde (MDA) levels. MDA, a lipid peroxidation end product, reacts with thiobarbituric acid (TBA) in an acidic medium (pH 3.5) to form a pink complex, which is measured at 532 nm. Reagents included 1.15% potassium chloride, 8.1% sodium dodecyl sulfate (SDS), 20% acetic acid, 0.8% TBA in 0.1N NaOH, a n-butanol/pyridine mixture (15:1 v/v), and 1,1,3,3-tetramethoxypropane (TMP) for standards. Here, reagents without the sample were used to measure background absorbance while Standard curve was Prepared using Tetramethoxypropane (TMP) as an MDA standard.

For the procedure, 0.1 ml of the sample was mixed with 0.2 ml of 8.1% SDS solution, followed by 1.5 ml of 20% acetic acid (pH 3.5), and 1.5 ml of 0.8% TBA solution. The total volume was adjusted to 4.0 ml with distilled water. This mixture was heated at 95 °C for 60 min, cooled, then mixed with 1.0 ml of distilled water and 5.0 ml of the n-butanol/pyridine mixture. After vigorous shaking and centrifugation at 4,000 rpm for 10 min, the organic layer was separated, and its absorbance was measured at 532 nm. A standard curve was created using different TMP concentrations.

In summary, 0.1 ml of the sample, TMP standards, and potassium chloride solution for the blank were each mixed with SDS, acetic acid, TBA, and distilled water to a final volume of 4.0 ml. The mixtures were heated, cooled, combined with distilled water and the n-butanol/pyridine mixture, shaken centrifuged, and the absorbance of the organic layer was recorded at 532 nm.

#### 2.5.5 Assay of super oxide dismutase

The SOD assay was performed using the Bovine Superoxide Dismutase ELISA kit by BT Lab (catalog no. E003Bo). To prepare the sperm homogenate, 2 mL of diluted semen containing 160 million sperm was centrifuged at 8,000 rpm for 15 min at 4 °C. The resulting pellet was resuspended in 0.01 M PBS and subjected to three freeze-thaw cycles in liquid nitrogen. The samples were then centrifuged at 10,000 rpm for 15 min, and the supernatant was collected, diluted 1:1 with PBS, and stored at −80 °C until further processing. Buffer without sample was used as Blank control. Sperm homogenate without enzyme activation was used as negative control and known SOD enzyme standard from the ELISA kit was used as positive control.

For the assay, all kit components and samples were brought to room temperature. The wash buffer was prepared by diluting the concentrate with distilled water. Standards, blanks, and samples (50 μL each) were added to their respective pre-coated ELISA plate wells, along with 50 μL of Biotinylated Antibody and the plate was incubated for 60 min at 37 °C. After incubation, the wells were washed, and 300 μL of wash buffer Three times. Substrate Solution A and B were added, followed by a 15-min incubation at room temperature in the dark. Afterward, 50 μL of stop solution was added, changing the color from blue to yellow. The optical density (OD) was measured at 450 nm within 10 min. A standard curve was created from the OD values of the standards, and the GSH-Px concentrations in the samples were determined by comparing their OD values to the standard curve.

#### 2.5.6 Assay of glutathione peroxidase

The SOD assay was performed using the Bovine Glutathione Peroxidase ELISA kit by BT Lab (catalog no. E0006Bo) in a manner similar to super oxide dismutase. Buffer without sample was used as blank control, heat-inactivated sperm homogenate was used as negative control, and known GPx enzyme standard from the ELISA kit was used as positive control.

#### 2.5.7 Assay of Reactive Oxygen Species (ROS)

ROS production in samples was assessed following the method of Falchi et al. (14). Each 25 μL sample was diluted in 1 mL PBS with 10 μM 2′, 7′-dichlorofluorescein diacetate (H2DCFDA) and incubated in the dark for 30 min at 38 °C. After incubation, samples were centrifuged at 4,229 rpm for 3 min, the supernatant was discarded, and the pellet was resuspended in 500 μL of 2% paraformaldehyde and kept at 4 °C for 1 h. Following fixation, samples were centrifuged again at 4,229 rpm for 3 min, the supernatant was removed, and the pellet was resuspended in 300 μL PBS. Samples were stored in the dark at 4 °C initially and then were stored at −80 °C until flow cytometric analysis within a month. ROS estimation using Chemiluminescence Assay has Blank control which included no sperm, only reagents to measure baseline luminescence.

For analysis, samples were excited with a blue laser (488 nm) and detected using the FITC channel. A total of 10,000 events were examined per sample. Doublets and clumps were excluded using a polygon gate for data accuracy, and the main population was gated under the histogram using Auto Line Segment. Spermatozoa were classified as ROS-positive or ROS-negative. Data were collected and analyzed using CytExpert software (v.2.3).

#### 2.5.8 Statistical analysis

The data was analyzed using the SPSS (Version 23) software package. Sperm quality parameters among means was tested by Duncan's multiple range tests. Significant differences were considered when *P* ≤ 0.05. The values for all parameters were given as mean ± standard error.

## 3 Results

### 3.1 Characterization of NP

**Figure 1:**
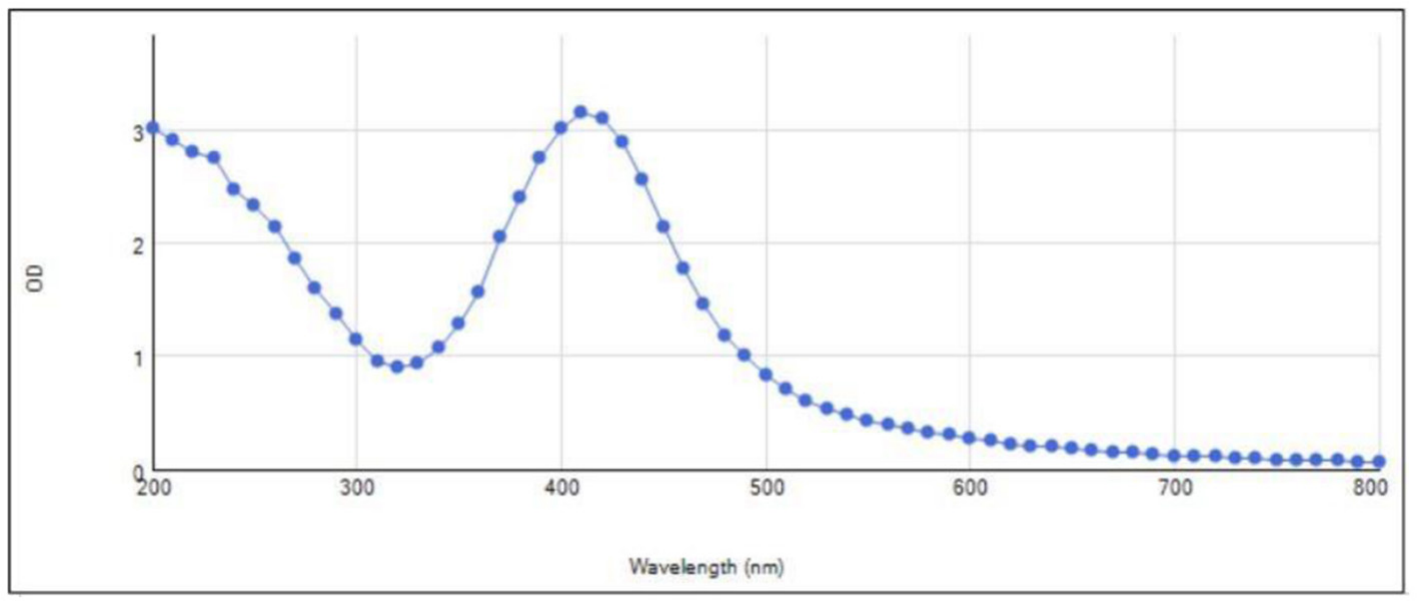
UV spectrophotometry of prepared nanoparticles.

The NP's were dried on hot plate and collected on a petri plate. The yield of NP's collected was 0.48 g. The UV-Vis absorption spectrum displayed confirms NP formation as characterized by two prominentabsorption peaks (Figure 1). The smaller peak is around 250–300 nm, which can be attributed to electronic transitions within the silver nanoparticles, signaling the presence of smaller particles and possibly some aggregated forms. The second peak, observed around 400–450 nm, is characteristic of the surface plasmon resonance (SPR) of silver nanoparticles.

**Figure 2:**
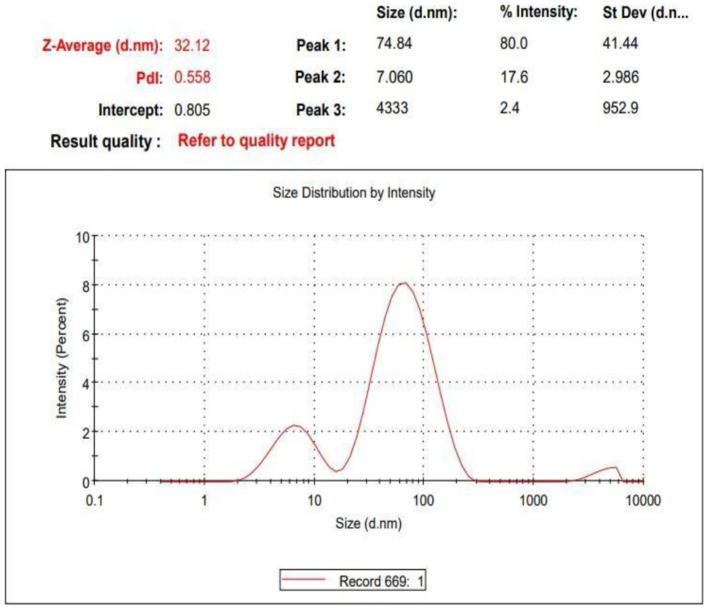
Z- average of nanoaprticles.

The Z-average size of the nanoparticles is 32.12 nm (Figure 2), indicating that the average diameter of the particles is around 32 nm. The polydispersity index (PdI) is 0.558, which suggests a moderate level of distribution uniformity in the sample. Ideally, a PdI closer to 0 indicates a narrow size distribution, while values above 0.5 suggest a broader distribution. In this case, a PdI of 0.558 indicates Some variability in particle sizes, though not excessive The size distribution graph shows a dominant peak at approximately 74.84 nm, accounting for 80% of the intensity, which indicates that most of the nanoparticles are concentrated around this size. A secondary peak appears at around 7.06 nm, contributing 17.6% of the intensity, suggesting the presence of a smaller fraction of nanoparticles, while a third peak at 4,333 nm is minor and likely represents larger aggregates or impurities in the sample.

**Figure 3:**
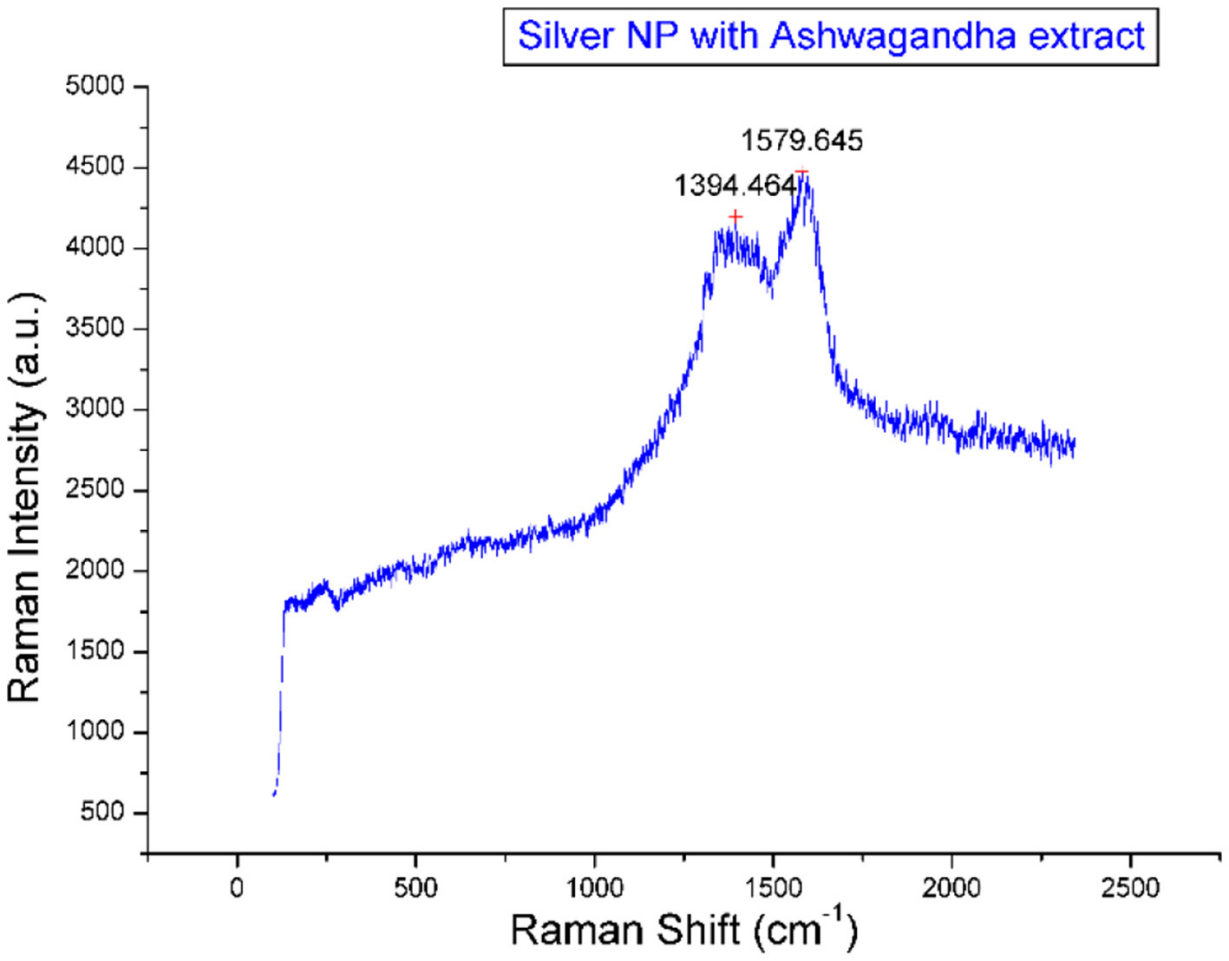
Raman spectrometry of prepared nanaoparticles.

The Raman spectroscopy spectrum of silver nanoparticles (AgNPs) synthesized with Ashwagandha extract displays characteristic peaks (Figure 3). The spectrum shows two prominent peaks at 1394.46 cm^−1^ and 1579.645 cm^−1^. These peaks are associated with the vibrational modes of organic compounds, specifically attributed to the stretching of C-H bonds in aromatic rings, which is likely influenced by the components of the Ashwagandha extract used during nanoparticle synthesis.

The peak at 1579.645 cm^−1^ corresponds to the D band, which is typically seen in carbon-based materials and indicates disorder or defects in the carbon structure, possibly due to the reduction of silver ions by the Ashwagandha extract. The 1394.46 cm^−1^ peak, corresponding to the G band, is associated with the graphitic nature of the material, indicating the presence of carbon structures.

The significant intensity observed at these peaks confirms the interaction between the silver nanoparticles and the biomolecules from the Ashwagandha extract, suggesting that the extract might act as both a reducing and stabilizing agent during the nanoparticle synthesis. This Raman analysis highlights the role of Ashwagandha in modifying the nanoparticle surface and providing chemical stability. Raman spectroscopy here indicates the presence of aromatic and graphitic carbon structures, most likely due to polyphenolic or flavonoid-type compounds from Ashwagandha acting as reducing/stabilizing agents in AgNP synthesis. However, identification of a specific molecule would require further analysis (e.g., HPLC, LC-MS, or NMR) in combination with the Raman spectrum.

**Figure 4:**
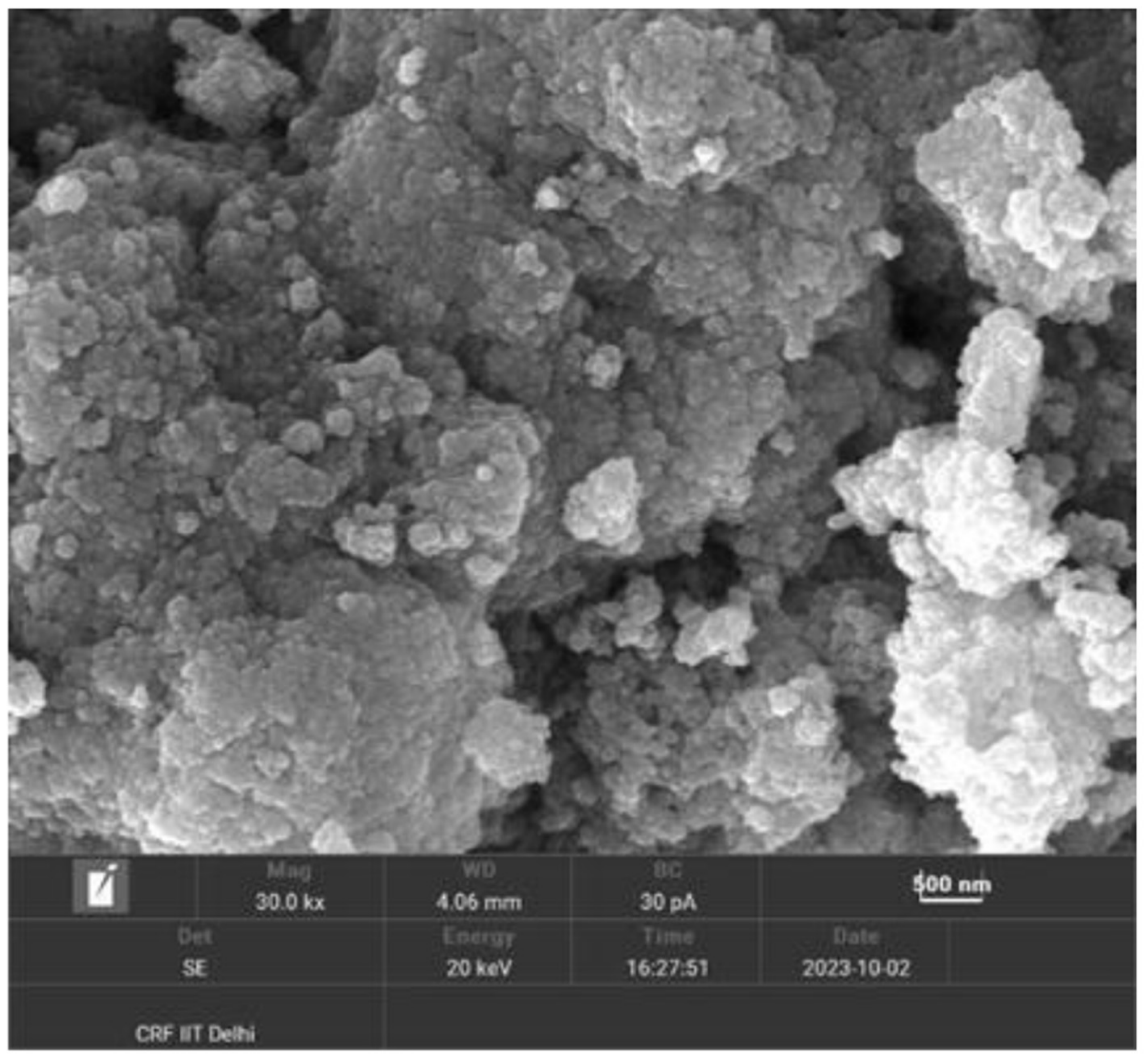
Scanning Electron Microscopy images of prepared nanoaprticles.

The AgNPs appear to be roughly spherical and exhibit a high degree of polydispersity, as evidenced by the varied sizes of the particles within the clusters. The surface morphology indicates that the nanoparticles are aggregated, forming large clusters rather than being dispersed individually. This aggregation might be due to van der Waals forces or other intermolecular interactions. Overall, the SEM image highlights the successful synthesis of silver nanoparticles with a significant degree of aggregation and varied particle sizes, which can be further analyzed for their specific applications and properties (Figure 4).

**Figure 5:**
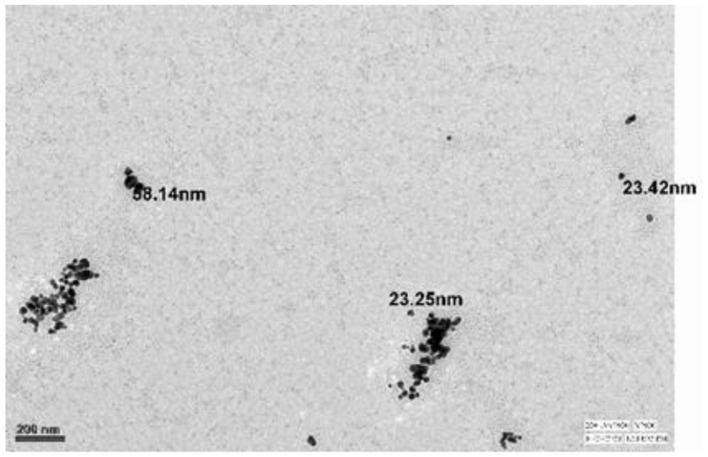
Transmission Electron Microscopy images of prepared nanoaprticles.

TEM: The particles appear to be roughly spherical, with smooth surfaces, which is typical for silver nanoparticles synthesized through various chemical and green synthesis methods. The uniform size and spherical shape of the AgNPs, along with their dispersion, suggest a successful synthesis process, with potential for high reactivity and surface area. These characteristics are desirable for applications requiring consistent nanoparticle performance. Figure 5 shows TEM image of NP.

**Figure 6:**
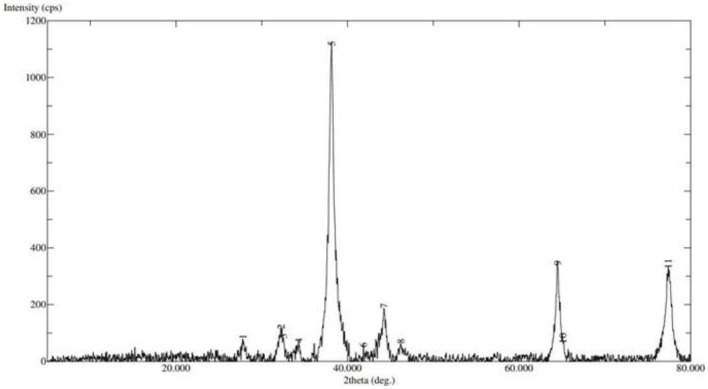
X-Ray Diffraction image of prepared nanoparticles.

XRD: The X-ray Diffraction (XRD) pattern of the silver nanoparticles (AgNPs) provides critical insight into their crystalline structure. The diffraction peaks observed in the XRD spectrum can be indexed to the face-centered cubic (fcc) structure of silver. The major peaks are located at approximately 2θ values of 38.1°, 44.3°, 64.5°, and 77.4°, corresponding to the (111), (200), (220), and (311) planes of silver, respectively (Figure 6).

The intense peak at 38.1° indicates that the (111) plane is the most densely packed and pre-dominan orientation in the synthesized AgNPs, suggesting a high degree of crystallinity. The sharpness and intensity of the peaks imply that the nanoparticles are well-crystallized. The absence of additional peaks indicate that the sample is relatively pure with no significant contamination or secondary phases. The narrow width of the diffraction peaks can be used to estimate the crystallite size of the nanoparticles using the Scherrer equation, which typically confirms the nanoscale dimensions of the synthesized AgNPs. The average crystallite size was estimated using the Debye–Scherrer equation: D = Kλ/βcosθ where D is the crystallite size, K is the shape factor (0.9), λ is the ray wavelength (0.15406 nm), β is the full width at half maximum (FWHM) in radians, and θ is the Bragg angle. Using the FWHM of the (111) peak (assumed as 0.8° for calculation), the average crystallite size was determined to be approximately 10.5 nm, confirming the nanoscale nature of the synthesized particles.

In summary, the XRD analysis confirms that the synthesized silver nanoparticles are crystalline with face-centered cubic structure, predominantly oriented along the (111) plane, and are of high purity. This crystalline nature is crucial for applications requiring high catalytic and antimicrobial activity.

### 3.2 2,2-diphenyl-1-picrylhydrazyl (DPPH) activity

The DPPH assay revealed that EA-AgNPs exhibited dose-dependent antioxidant activity. At lower concentrations (10 μg/mL), the scavenging activity was modest (6.95%), while at higher concentrations (80 μg/mL), the activity reached 38.95%. This increase suggests that EA-AgNPs have potential as effective antioxidants. Table 1 shows absorbance and scavenging activity (%) of different concentrations of NP.

**Table 1:** Absorbance and scavenging activity (%) of different concentrations of NP.

**NP concentration**	**Absorbance (OD)**	**Scavenging activity %**
A10	0.375	6.949
A20	0.363	9.9255
A30	0.356	11.625
A40	0.349	13.40
A60	0.335	16.87
A80	0.246	38.95

### 3.3 Selection of optimum concentration of Ethanolic Ashwagandha-Silver Nanoparticles (EA-AgNPs):

In the study assessing the optimal concentration of EA-AgNPs for semen supplementation, ten ejaculates were divided into five groups, with the control group receiving no supplementation and the others receiving 10, 20, 30, and 40 μg/mL of EA-AgNPs. Results indicated a slight improvement inmotility at 10 and 20 μg/mL (71.81 ± 1.58 and 71.89 ± 1.61, respectively) compared to the control (70.36 ± 1.44), while higher concentrations (30 and 40 μg/mL) led to a decrease in motility, particularly at 40 μg/mL (63.73 ± 2.718). The bent tail percentage was significantly lower at 10 and 20 μg/mL (0% incidence), with increases at higher concentrations. Other parameters, including sperm head diameter, curvilinear velocity, and straight-line velocity, showed no significant differences across groups. Overall, 10 and 20 μg/mL were identified as the best concentrations for maintaining motility and minimizing adverse effects, with 20 μg/mL being the optimal choice. The CASA data is shown in Tables 2–4.

**Table 2:** Effect of different concentrations of Ethanolic Ashwagandha-Silver Nanoparticles (EA-AgNPs) on sperm motility and abnormality parameter.

**Parameter**	**Group 1 control (without NP)**	**Group 2 (EA-AgNP @10 μg/mL)**	**Group 3 (EA-AgNP @20 μg/mL)**	**Group 4 (EA-AgNP @30 μg/mL)**	**Group 5 (EA-AgNP @40 μg/mL)**	***P*-value**
Motile (%)	70.36 ± 1.44^ab^	71.81 ± 1.58^b^	71.89 ± 1.61^b^	68.77 ± 2.78^ab^	63.73 ± 2.718^a^	0.05
Progressive motile (%)	53.64 ± 2.82	52.52 ± 5.22	54.08 ± 4.79	52.26 ± 4.64	46.22 ± 3.69	0.70
MotileConc. (Million/ml)	7.80 ± 1.41	9.26 ± 1.11	8.56 ± 1.32	9.66 ± 1.15	8.89 ± 1.37	0.87
Motile mean area (μm^2^)	18.13 ± 0.51	17.78 ± 0.29	18.49 ± 0.50	18.60 ± 0.41	18.62 ± 0.37	0.57
DMR (%)	2.35 ± 0.44	2.37 ± 0.52	2.6 ± 0.87	3.56 ± 1.02	2.91 ± 0.63	0.76
Distal droplet (%)	0	0.03	0	0	0	–
Bent tail (%)	0.47 ±0.28^ab^	0.000^a^	0.000^a^	0.06 ± 0.06^a^	0.95 ± 0.15^b^	0.014

Mean values with different superscript in a row differ significantly (*p* ≤ 0.05).

**Table 3:** Effect of different concentrations of Ethanolic Ashwagandha-Silver Nanoparticles (EA AgNPs) on the actual kinematic parameters.

**Parameter**	**Group 1 control (without NP)**	**Group 2 (EA-AgNP @10 μg/mL)**	**Group 3 (EA-AgNP @20 μg/mL)**	**Group 4 (EA-AgNP @30 μg/mL)**	**Group 5 (EA-AgNP @40 μg/mL)**	***P*-value**
DAP μm	18.41 ± 0.61	18.68 ± 0.79	18.70 ± 1.04	18.33 ± 1.06	17.40 ± 0.70	0.82
DCL μm	33.67 ± 0.89	32.73 ± 1.26	32.93 ± 1.62	32.71 ± 1.97	32 ± 0.95	0.94
DSL μm	15.56 ± 0.63	16.02 ± 0.75	15.83 ± 0.92	15.33 ± 0.93	14.23 ± 0.65	0.53
ALH μm	7.79 ± 0.25	7.33 ± 0.51	7.33 ± 0.49	7.38 ± 0.039	7.75 ± 0.027	0.84
BCF Hz	24.51 ± 0.61	23.66 ± 0.38	23.55 ± 0.60	24 ± 0.64	23.66 ± 0.72	0.43
VAP μm/s	97.37 ± 3.42	93.87 ± 6.68	95.23 ± 6.67	91.62 ± 5.28	92.56 ± 5.56	0.95
VCL um/s	174.02 ± 5.03	164.09 ± 11.51	165.31 ± 11.15	161.37 ± 10.18	165.78 ± 8.09	0.90
VSL um/s	84.37 ± 3.48	81.91 ± 6.11	82.68 ± 5.91	78.16 ± 4.62	78.91 ± 5.10	0.90

Mean values with different superscript in a row differ significantly (*p* ≤ 0.05).

**Table 4:** Effect of different concentrations of Ethanolic Ashwagandha-Silver Nanoparticles (EA-AgNPs) on the relative kinematic parameters.

**Parameter**	**Group 1 control (without NP)**	**Group 2 (EA-AgNP @10 μg/mL)**	**Group 3 (EA-AgNP @20 μg/mL)**	**Group 4 (EA-AgNP @30 μg/mL)**	**Group 5 (EA-AgNP @40 μg/mL)**	***P*-value**
STR %	85.59 ± 1.15	87.55 ± 1.10	86.89 ± 0.99	85.31 ± 1.05	84.26 ± 1.44	0.29
LIN %	49 ± 1.17	51.48 ± 2.04	51.18 ± 1.42	49.78 ± 1.19	47.97 ± 0.99	0.37
WOB %	56.56 ± 0.81	58.60 ± 1.78	58.49 ± 1.09	58.01 ± 00.89	56.30 ± 0.73	0.45

### 3.4 Freezing of semen supplemented with optimum concentration of EA-AgNPs

The data compares various sperm parameters between the control group (Group 1, without NP supplementation) and the nanoparticle-supplemented group @ 20 μg/mL (Group 2) before and after freezing along with their respective statistical analyses. Group 2 exhibited significantly higher percentages of motile sperm and progressive motility before freezing, but no significant differences were observed after freezing. The motile sperm concentration was significantly higher in Group 2 before freezing, but not after.

No significant differences were found in distal midpiece reflex, distal droplet percentage, and bent tail percentage between the groups. Group 2 showed significantly higher amplitude of lateral head displacement (ALH) before freezing, while Group 1 had higher beat cross frequency (BCF). After freezing, Group 1 had significantly higher distance curved line (DCL), but no significant differences were observed in other parameters. NP supplementation selectively enhanced specific pre-freezing kinematic parameters such as ALH, while its impact on post-freezing sperm motility was limited.

Cryopreservation diminished the differences between the groups, reflecting its broader effects on sperm motility. No significant differences were found in straightness (STR), linearity (LIN), and wobble (WOB) before freezing. After freezing, Group 2 showed higher STR, indicating improved sperm straightness with NP supplementation, while LIN and WOB showed minimal differences. NP supplementation had a protective effect on STR during cryopreservation, potentially improving post- thaw sperm straightness and maintaining motility quality, but had minimal effects on LIN and WOB. The results are showed in Tables 5–7.

**Table 5:** Effect of different concentrations of Ethanolic Ashwagandha-Silver Nanoparticles (EA-AgNPs) @ 20 μg/mL on sperm motility and abnormality parameters before and after freezing.

**Parameter**	**Cryopreservation status**	**Group 1 (without NP)**	**Group 2 (EA- AgNPs @ 20 μg/mL)**	***T*-value**	***P*-value**
Motile % of total	Before freezing	66.18^a^ ± 1.30	72.15^b^ ± 0.70	−3.64	0.0002
	After freezing	56.20^a^ ±2.36	60.83^b^ ±1.50	−1.41	0.10
Progressive % of total	Before freezing	50.43^a^ ± 1.82	56.65^b^ ± 1.13	−2.41	0.009
	After freezing	49.38 ±2.90	51.27 ±2.86	−0.69	0.64
Motile concentration (million/ml)	Before freezing	43.93^a^ ± 1.05	47.70^b^ ± 0.66	−2.73	0.004
	After freezing	38.60 ± 2.36	39.33 ± 2.51	−0.08	0.83
DMR %	Before freezing	2.75 ± 0.214	2.31 ± 0.242	1.16	0.12
	After freezing	2.93 ± 0.83	2.30 ± 0.31	0.74	0.47
Distal droplet %	Before freezing	0.061 ± 0.38	0.027 ± 0.018	0.69	0.24
	After freezing	0.468 ± 0.060	0.509 ± 0.09	−0.047	0.70
Bent tail (%)	Before freezing	0.192 ± 0.06	0.065 ± 0.04	1.69	0.178
	After freezing	0.152 ± 0.03	0.148 ± 0.03	0.16	0.94

Mean values with different superscript in a row differ significantly (*p* ≤ 0.05).

**Table 6:** Effect of different concentrations of Ethanolic Ashwagandha-Silver Nanoparticles (EA-AgNPs) @ 20 μg/mL on the actual kinematic parameters before and after freezing.

**Parameter**	**Cryopreservation status**	**Group 1 (without NP)**	**Group 2 (EA- AgNPs @ 20 μg/mL)**	***T*-value**	***P*-value**
DAP	Before freezing	18.40 ± 0.50	18.00 ± 0.36	0.88	0.18
	After freezing	32.90 ± 1.00	30.44 ± 1.12	1.45	0.10
DCL	Before freezing	31.75 ± 0.54	31.64 ± 0.55	0.09	0.46
	After freezing	54.28^b^ ± 2.10	46.90^a^ ± 2.41	2.11	0.02
DSL	Before freezing	15.79 ± 0.50	15.32 ± 0.357	1.10	0.136
	After freezing	28.65 ± 1.00	27.56 ± 0.95	0.59	0.43
ALH	Before freezing	6.96^a^ ± 0.16	7.53^b^ ± 0.18	−2.29	0.02
	After freezing	5.06 ± 0.428	5.54 ± 0.42	−2.40	0.12
BCF	Before freezing	25.15^b^ ± 0.36	24.02^a^ ± 0.43	1.87	0.03
	After freezing	27.08 ± 0.94	27.38 ± 0.70	−0.29	0.79
VAP	Before freezing	92.09 ± 2.17	94.53 ± 1.51	−0.86	0.19
	After freezing	90.41± 3.97	90.13^b^ ± 3.50	−1.72	0.95
VCL	Before freezing	157.81 ± 3.95	163.53 ± 3.18	−1.27	0.10
	After freezing	150.20 ± 5.28	147.72 ± 6.47	−2.24	0.77
VSL	Before freezing	81.52 ± 1.99	82.85 ± 1.49	−0.38	0.35
	After freezing	86.482 ±3.88	85.98 ±2.59	1.10	0.91

Mean values with different superscript in a row differ significantly (*p* ≤ 0.05).

**Table 7:** Effect of different concentrations of Ethanolic Ashwagandha-Silver Nanoparticles (EA-AgNPs) @ 20 μg/mL on the relative kinematic parameters before and after freezing.

**Parameter**	**Cryopreservation status**	**Group 1 (without NP)**	**Group 2 (EA- AgNPs @ 20 μg/mL)**	***T*-value**	***P*-value**
STR %	Before freezing	87.35 ± 0.99	87.20 ± 0.63	0.47	0.32
	After freezing	87.40^a^ ± 1.34	91.47^b^ ± 0.88	−2.544	0.01
LIN	Before freezing	52.35 ± 1.28	51.27 ± 0.88	1.02	0.15
	After freezing	57.63^a^ ± 2.11	59.30^b^ ± 2.29	−2.65	0.49
WOB	Before freezing	58.16 ± 0.73	58.46 ± 0.67	0.85	0.20
	After freezing	60.79 ± 1.22	61.42 ±1.87	−2.58	0.70

Mean values with different superscript in a row differ significantly (*p* ≤ 0.05).

### 3.5 Assessing lipid peroxidation, acrosome integrity, and ROS levels in cryopreserved semen

The Table 8 provides a comparison of biochemical parameters related to sperm quality between Group 1 (unsupplemented) and Group 2 (NP-supplemented), focusing on lipid peroxidation, intact acrosome, reactive oxygen species (ROS), and antioxidant enzymes such as superoxide dismutase (SOD) and glutathione peroxidase (GPx).

**Table 8:** Lipid peroxidation, acrosome integrity, Reactive Oxygen Species (ROS), Super Oxide Desmutase (SOD), and Glutathion Peroxidase (GPx) levels in semen sample.

**Biochemical parameters**	**Group 1**	**Group 2**
Lipid peroxidation (n mol MDA)	460.88 ± 961.70	305.324 ± 575.60
Intact acrosome	68.83^b^ ± 1.35	71.50^a^ ± 1.47
Reactive Oxygen Species (ROS)%	3.4 ± 1.17	1.05 ± 0.48
Super Oxide Desmutase (SOD) ng/mL	26.656 ± 4.0732	28.813 ± 13.1920
Glutathion (GPx) ng/mL	38.52 ± 0.0090	38.74 ± 0.0100

Mean values with different superscript in a row differ significantly (*p* ≤ 0.05).

Lipid peroxidation, measured as malondialdehyde (MDA) levels, was lower in the NP-supplemented group (305.324 ± 40.70 nmol/mL) compared to the unsupplemented group (460.88 ± 68.00 nmol/mL), though this difference was not statistically significant (*P* = 0.12). This suggests a potential trend toward reduced oxidative damage in the supplemented group.

Intact acrosome percentage, a crucial marker for sperm functionality, was significantly higher in the NP-supplemented group (71.50 ± 1.47%) compared to the unsupplemented group (68.83 ± 1.35%; *P* = 0.001). This indicates that NP supplementation helps maintain acrosomal integrity, which is vital for successful fertilization.

Reactive oxygen species (ROS) levels were lower in the NP-supplemented group (1.05 ± 0.48%) compared to the unsupplemented group (3.4 ± 1.17%), though the difference was not statistically significant (*P* = 0.13). This reduction aligns with the potential antioxidant effects of NP supplementation.

For antioxidant enzymes, no significant differences were observed between the groups. SOD level were comparable (22.82 ± 0.0004 ng/mL in Group 1 vs. 22.78 ± 0.0012 ng/mL in Group 2; *P* = 0.76), and GPx levels were also similar (38.52 ± 0.0090 ng/mL in Group 1 vs. 38.74 ± 0.0100 ng/mL in Group 2; *P* = 0.87). These findings suggest that NP supplementation does not significantly alter the activity of these specific antioxidant enzymes.

Overall, NP supplementation appears to enhance sperm quality by improving acrosomal integrity and potentially reducing oxidative damage, as indicated by lower ROS levels and lipid peroxidation trends, although its effects on enzymatic antioxidant defenses remain negligible.

## 4 Discussion

The present study demonstrates the successful green synthesis of silver nanoparticles (AgNPs) using *Withania somnifera* (Ashwagandha) extract as a reducing and stabilizing agent. The use of plant extracts offers a sustainable, non-toxic alternative to conventional chemical synthesis methods. The phytochemicals in Ashwagandha, such as flavonoids, terpenoids, and reducing sugars, play a dual role facilitating the reduction of Ag^+^ ions to Ag° and stabilizing the formed nanoparticles, thereby preventing aggregation. These findings align with previous reports where plant-derived biomolecules enabled effective synthesis of stable and biologically active nanoparticles (15, 16). Oxidative stress damages sperm function by damaging the sperm membrane through lipid peroxidation, impairing the acrosome reaction and motility, and damaging DNA and proteins. This occurs because sperm have a high content of polyunsaturated fatty acids (PUFAs) in their membranes, making them vulnerable to reactive oxygen species (ROS), and possess limited antioxidant and DNA repair mechanisms. The damage to the plasma membrane, acrosome, and DNA leads to decreased sperm function and fertility. Reactive oxygen species (ROS) produced as a result of oxidative stress primarily originate from two locations: first, from the ubiquinone binding sites within complexes I and III of the electron transport chain of compromised mitochondria, and second, via the NOX system located in the sperm plasma membrane, which oxidizes sperm proteins and disrupts the covalent bonds of sperm DNA (17, 18). Silver nanoparticles (AgNPs) have a dual effect on cattle sperm, with low concentrations potentially reducing oxidative stress by decreasing reactive oxygen species (ROS) and lipid peroxidation, while high concentrations are detrimental, causing oxidative damage, apoptosis, and a decline in sperm quality (19). It has been seen that Quercetin-coated CuO–ZnO nanoparticles enhance post-thaw quality of ram semen. Quercetin-coated CuO ZnO nanoparticles at the dose rate of 5 μg/mL significantly improved sperm viability (74.86 ± 0.75), total motility (71.79 ± 1.07), membrane functionality (72.99 ± 1.17), and DNA integrity (95.79 ± 0.98), and reduced malondialdehyde levels (21.89 ± 0.17) (20). The 25 μM curcumin NPs when supplemented in semen showed significantly higher sperm motility (total motility: 72.67 ± 1.15 % and progressive motility: 59.2 ± 0.96 %) (21).

Silver sulfate was used to prepare NP's for this study as silver nitrate is unstable and can be toxic to tissues (22) &, Ag sulfate NPs show less toxicity to eukaryotic cells (23). Alkaline conditions (pH > 7) increase the reaction rate, which speeds up the conversion of silver ions (Ag+) into metallic silver (Ag°)nanoparticles. Higher pH values generally lead to smaller particle sizes due to an increased nucleation and growth rate, which is desirable for many applications. In green synthesis, the pH can affect the functional groups of biomolecules used for reduction and capping. A pH of 9 can optimize the activity of these functional groups, ensuring an effective reduction process (24, 25).

The biosynthesized AgNPs exhibited a characteristic brown coloration, attributed to surface Plasmon resonance (SPR), confirmed by UV-Vis spectroscopy with a strong absorption peak at 434 nm. SPR is sensitive to particle size, shape, and degree of aggregation, with red shifts indicating larger or aggregated particles. In comparison, previously reported SPR peaks for AgNPs synthesized using *Azadirachta indica, Mangifera indica*, and *Eucalyptus* also fall within this range (26, 27).

The synthesized AgNPs were further characterized by SEM and TEM, revealing Predominantly spherical particles ranging from ~4.7 to 32 nm. Minor shape irregularities and limited clustering suggest a degree of anisotropy, commonly observed due to solvent evaporation or interparticle interactions during sample preparation (28). The zeta potential measurement (−22.8 mV) indicated moderate stability of the colloidal suspension, as electrostatic repulsion among negatively charged particles helped prevent agglomeration (29).

XRD analysis confirmed the crystalline nature of AgNPs, showing intense peaks corresponding to the (111), (200), (220), and (311) planes, characteristic of a face-centered cubic (fcc) structure. The dominant (111) reflection suggests a high degree of crystallinity and preferred growth orientation, corroborating the findings of Saleh and Mahdi (34). The average crystallite size, calculated using the Debye–Scherrer equation, was approximately 10.5 nm, supporting the nanoscale dimensions observed under TEM.

FTIR analysis provided insight into the functional groups involved in nanoparticle stabilization. Peaks around 1,642 cm^−1^ indicated the presence of amine and carbonyl groups, likely derived from proteins or terpenoids acting as capping agents (30, 31). Raman spectroscopy further confirmed the interaction between silver and biomolecules, with characteristic cm^−1^ attributed to C–S–C, C=O symmetric and antisymmetric stretching vibrations, respectively (16, 32).

The antioxidant activity of the AgNPs, evaluated through DPPH radical scavenging assay, showed a concentration-dependent increase in activity, from 6.95% at 10 μg/mL to 38.95% at 80 μg/mL. This confirms the electron donating capacity of the nanoparticles, attributed to residual bioactive compounds from the Ashwagandha extract. The trend aligns with prior observations that antioxidant efficacy correlates with nanoparticle concentration and surface chemistry (40, 41).

From a reproductive biotechnology perspective, supplementation of cryopreserved semen with AgNPs synthesized using Ashwagandha extract showed concentration-dependent effects. Optimal concentrations (10–20 μg/mL) maintained or improved sperm motility and morphology, while higher doses (40 μg/mL) negatively impacted motility and increased tail abnormalities. These results suggest a therapeutic threshold beyond which nanoparticles may exhibit cytotoxicity, as reported in previous studies on metallic nanoparticles in semen preservation (38, 39).

Similar trends have been observed with other green-synthesized nanoparticles, such as gold, copper oxide, and thymoquinone-based nanomaterials, which improved sperm viability, motility, and antioxidant status at specific concentrations (36, 37). Our findings highlight the importance of optimizing nanoparticle concentration to harness beneficial effects while minimizing toxicity.

## 5 Conclusion

This study confirms that *Withania somnifera* (ashwagandha) extract provides an effective and eco- friendly method for the green synthesis of silver nanoparticles (AgNPs). The phytochemicals in the extract successfully reduced silver ions and stabilized the nanoparticles. However, the optimal concentration of nanoparticles varies depending on their type and application. While these findings highlight promising advancements, further research is required to establish standardized guidelines for nanoparticle use across different species and contexts, given the variability in outcomes depending on the specific nanoparticles and concentrations employed (35). Characterization analyses— including UV–visible spectroscopy, SEM, TEM, Raman spectroscopy, and XRD—verified the formation of predominantly spherical AgNPs. TEM imaging showed particle sizes mainly between 5 and 40 nm, with an average size of 32 nm, while the polydispersity index (PdI) of 0.558 indicated moderate size distribution. Zeta potential measurements revealed a negative surface charge, confirming good dispersion stability. Raman spectroscopy displayed two prominent peaks at 1394.46 cm^−1^ (G band) and 1579.645 cm^−1^ (D band), indicating the presence of aromatic and graphitic carbon structures from the ashwagandha extract and confirming its dual role as reducing and capping agent. XRD analysis showed characteristic diffraction peaks at 38.1°, 44.3°, 64.5°, and 77.4°, corresponding to the (111), (200), (220), and (311) planes of face-centered cubic silver. The intense (111) peak and narrow peak widths indicated high crystallinity, and the crystallite size calculated using the Debye–Scherrer equation was approximately 10.5 nm.

In this study, we observed that silver nanoparticles (AgNPs) significantly improve semen quality prior to freezing. However, the protective effects of AgNPs were positive, though insignificantly.

Cryopreservation subjects spermatozoa to a range of detrimental stressors, including membrane phase transition, oxidative stress, and mitochondrial damage. Each of these stressors plays a critical role in sperm viability post-thaw. The process of freezing and thawing induces changes in the lipid bilayer structure of sperm membranes, resulting in a phase transition from a liquid crystalline phase to a more rigid gel-like state. This transition disrupts membrane integrity, reducing sperm motility and viability. AgNPs have been shown to interact with lipid membranes, potentially stabilizing their structure prior to freezing, which could explain the observed improvements in semen quality before freezing. However, this stabilization may be insufficient to counteract the severe membrane damage caused by phase transitions during thawing, leading to a loss of AgNP protective effects post-thaw.

Acrosomal and Mitochondrial Damage: Cryopreservation is also known to induce acrosomal and mitochondrial dysfunction. The acrosome reaction, which is essential for fertilization, can be impaired by freeze-thaw cycles, and mitochondrial damage can reduce ATP production, further compromising sperm motility and overall functionality. AgNPs have demonstrated some ability to stabilize mitochondrial function under certain conditions, yet the cryopreservation process may overwhelm these protective effects. However, LPO & ROS levels were found to be reduced, while intact acrosome were found to be more in cryopreserved group.

Upon thawing, AgNPs may aggregate or change their physical properties due to changes in the temperature and the surrounding cryoprotectant medium. This aggregation could reduce the effective surface area of AgNPs available for interaction with sperm membranes and other cellular structures, thus impairing their ability to mitigate cryopreservation-induced damage. After thawing, the dynamics of AgNPs within the sperm environment may change. The interaction between AgNPs and the sperm cells may be altered due to shifts in pH, ionic strength, or protein binding. This reduced bioavailability could contribute to the loss of the protective effects of AgNPs post-thaw. Another possibility is that the spermatozoa may become overloaded with AgNPs during the pre-freezing treatment, leading to toxic effects during thawing. The interaction between AgNPs and the sperm membrane could cause disruption that, while protective during freezing, may become detrimental upon thawing, when the membrane is more vulnerable to oxidative stress and phase transitions. Thus, nanoparticle aggregation, reduced bioavailability of AgNPs:, cellular overload might be the reason for non-significant improvement in motility parameters of cryopreserved semen.

The synthesized AgNPs exhibited strong dose-dependent antioxidant activity in DPPH assays, demonstrating their potential as bioactive agents. Their application in semen evaluation further suggested beneficial effects on sperm quality and motility, highlighting their promise in reproductive biotechnology. Overall, this research underscores green synthesized AgNPs as multifunctional nanomaterials with valuable applications in biomedical science, agriculture, and reproductive health.

Continued investigations are recommended to optimize nanoparticle stability and explore wider practical uses across various scientific and industrial domains.

## Data Availability

The original contributions presented in the study are included in the article/supplementary material, further inquiries can be directed to the corresponding author.
